# Educational program for patients with type-1 diabetes mellitus receiving free monthly supplies of insulin improves knowledge and attitude, but not adherence

**DOI:** 10.4103/0973-3930.44079

**Published:** 2008

**Authors:** R. Vimalavathini, S. M. Agarwal, B. Gitanjali

**Affiliations:** Department of Pharmacology, Jawaharlal Institute of Post Graduate Medical Education and Research (JIPMER), Pondicherry - 605 006, India

**Keywords:** Adherence, attitude, knowledge, practice, type-1 diabetes mellitus

## Abstract

**BACKGROUND:**

Though patients attending a diabetic clinic in a tertiary care hospital were given free monthly supplies of insulin, it was found that their glycemic control was poor.

**SETTINGS AND DESIGN:**

A prospective interventional study was carried out at the outpatient clinic in a tertiary care hospital.

**AIMS:**

To evaluate the effectiveness of a six month educational interventional program on the knowledge, attitude and practices (KAP) of type-1 diabetic patients receiving free monthly supplies of human insulin and to assess their adherence.

**METHODOLOGY:**

Sixty-seven type-1 diabetics, receiving free insulin vials each month, were recruited. The patients' baseline glycemic index, plasma insulin and KAP scores were determined using a validated questionnaire. The patients were educated about the disease and use of insulin for the next six months. In the seventh month, the KAP questionnaire was readministered and blood parameters measured.

**STATISTICAL ANALYSIS:**

Blood glucose, glycosylated hemoglobin and plasma insulin were compared by paired t tests. Mean KAP scores by Wilcoxon matched-pairs signed-ranks test. Difference in the proportion of patients answering the items was compared using test of proportions for dependant groups.

**RESULTS:**

The overall mean scores (± SE) increased from 30.8 ± 0.5 to 42.2 ± 0.4 (*P* < 0.001). The improvement in practice scores, though significant, was marginal, that is, from 17.7 ± 0.3 to 18.8 ± 0.3. In three out of the ten items under practice domain, only the manner in which vials were being stored at home showed significant improvement (*P* < 0.0001). The adherence to the insulin regimen increased from 82 to 86%, but was not significant. Patients cited financial reasons for nonadherence.

**CONCLUSION:**

The study showed that a planned educational intervention in type-1 diabetics, receiving monthly supplies of insulin free of charge, did not improve the key aspects of the practice component, even though the knowledge and attitude improved.

## Introduction

Adherence, a critical component in the treatment of chronic diseases like diabetes, is the extent to which patients' activities such as taking medication or changing the lifestyle coincides with medical advice. If the patient understands the pathophysiology of diabetes and the process involved in the management and treatment of diabetes, his degree of compliance will improve leading to better glycemic control.[1] Insulin remains the mainstay of treatment for type-1 diabetes mellitus and to some extent for type-2 diabetes as well. Nonadherence to the insulin regimen is a major hindrance in achieving the treatment goal,[2] since the insulin regimen itself is costly, painful and difficult.[3]

When a patient does not respond to an appropriately prescribed medicine, the reasons could be due to drug or patient-related factors. The patient-related factors fall into three categories – pharmionics, pharmacodynamics and pharmacokinetics. Pharmionics is the discipline concerned with what patients do with prescribed drugs.[4] Pharmionic factors contributing to lack of efficacy of a drug includes failure of taking the medicine in the prescribed doses, at the specific times and in keeping-up with other dosing instructions that are needed for satisfactory therapeutic action.[5] Hence, satisfactory pharmionics practice on the part of the patient is essential for optimal pharmacokinetic and pharmacodynamic action of the drug.

Planned interventional education programs have shown to provide a positive impact on improving the KAP scores in diabetic patients.[6] Prior to starting any educational program, it has been found appropriate to gauge the awareness level of the community under study by conducting a KAP study. This will help in implementing more successful health education programs by tailoring it to the needs of that particular community with improved adherence as one of the outcomes.[7]

The present study was a clinic-based descriptive study conducted at the diabetic clinic in the outpatient department (OPD) of the Jawaharlal Institute of Postgraduate Medical Education and Research (JIPMER), Puducherry, where patients come to collect their monthly supplies of human insulin free of cost. A blood sample measuring their random blood glucose revealed that 77 out of 131 patients were hyperglycemic, indicating that their glycemic control was poor despite receiving free monthly supplies of insulin. Since it has been shown that the KAP of diabetic patients are inadequate in India,[8] it was hypothesized that a planned interventional program of educating the patients on achieving good glycemic control will improve their glycemic status. Therefore, the objectives of the present study were to evaluate the KAP of diabetes mellitus among the type-1 diabetic patients receiving free monthly supplies of human insulin. We also planned to assess their adherence and evaluate the effectiveness of the planned interventional program for a period of six months, during the course of which the patients would be educated on how to achieve good glycemic control.

## Materials and Methods

### Patients

Sixty seven registered type-1 diabetic patients, of either gender, >18 years of age, visiting JIPMER diabetic clinic in the OPD to receive free human insulin vials on a monthly basis were recruited for the study. The study was conducted from May to December 2006. Those taking oral hypoglycemic agents or any other drugs which interfered with blood glucose values were excluded from the study. Also, patients with underlying liver and/or kidney disease, those collecting insulin by proxy and/or those who had not attended the diabetic clinic for at least two months were excluded from the study. The study was approved by the Institute Ethics Committee. Written informed consent was obtained from each patient.

### Data collection

Data regarding the patient's demographic characteristics and KAP were collected by individually interviewing the patients using a questionnaire. General characteristics included age, gender, occupation, monthly income, educational status, family history of diabetes, duration of the disease, type of insulin taken and hospital admission, if any, with the reason for admission and duration of stay. Anthropometric variables measured were height, weight and waist circumference.

### Development and validation of the questionnaire

The questionnaire consisted of three domains, that is, knowledge, attitude and practice. Knowledge reflects the extent to which the patient understands the disease, attitude reflects their beliefs and practice reflects how they put their knowledge and attitude into action. Only the knowledge, attitude and practice part of the questionnaire was scored. Knowledge had eight questions regarding the signs, symptoms and complications of diabetes, timing and dosing of insulin and effects of injecting too much or too little insulin. Attitude had four questions regarding the disease, drug, life style and their inclination to know more about the disease and its management. Practice had ten questions regarding storage of insulin, life style and dietary habits, withdrawal of insulin in the syringe, frequency of monthly visit to receive insulin and frequency of testing blood glucose. The items for the questionnaire were constructed from published and validated questionnaires;[8,9] and relevant questions for our study was also included based on expert opinion. The questionnaire was then administered to 100 type-1 diabetic patients to assess the relevance and suitability of the questionnaire in these patients. The questionnaire was refined accordingly and was readministered. The total score was 60. Cronbach's alpha was calculated and showed good internal consistency (*r* = 0.72).

### Definitions and categorical cut points Characteristics

Patients with a body mass index (BMI) of <19 were considered underweight, 19–25 as normal and 26–30 as overweight. A waist circumference of <35 inches in women and <40 inches in men was considered normal. Education level was graded as those who had never attended school, had primary, secondary, or tertiary education. Occupation status was recorded as student, unemployed (which included housewives), nonprofessional, professional and pensioner. Patients with a monthly income of <Rs. 2,500 were considered to belong to economically weaker sections, Rs. 2,501–5,500 to low income group, Rs. 5,501–Rs. 10,000 to middle income group and more than Rs.10,000 to high income group.[10]

#### Metabolic parameters

A fasting blood glucose of <130 mg/dL and a post prandial blood glucose of <180 mg/dL was considered as good glycemic control. HbA1c level of >7% was taken to denote poor glycemic control.[11] A C-peptide level of <0.3 pmol/ml was regarded as negligible endogenous insulin secretion.[12] Adherence to insulin was defined as those patients with plasma insulin levels of at least 50% of their previous insulin dose.

## Methodology

Blood samples were collected from the patients and on the same day the KAP questionnaire was administered. Liver and kidney functions tests and plasma blood glucose was determined with the commercially available kits using the autoanalyser. HbA1c was measured by using commercially available ion exchange chromatography kits. Plasma insulin and C-peptide were measured using immunoassay kits.

Education was in a classroom setting with 2–3 patients at a time. The patients were educated about the disease, its complications, foot care, sick-day management, hypoglycemia, drug and its storage using flip charts and pamphlets each month for the next six months. The need for maintaining near glycemic control was also stressed. In the seventh month, the KAP questionnaire was readministered and blood parameters were measured again.

### Statistics

Levels of blood glucose, glycosylated hemoglobin, plasma insulin and insulin antibodies measured before and after the intervention were compared by two-tailed paired *t* tests. Mean KAP scores between the baseline and after the intervention by Wilcoxon matched-pairs signed-ranks test. Any change in the percentage of patients answering correctly before and after the educational intervention was compared using test of proportions for dependant groups. For all statistical analyses, a *P*-value of <0.05 was considered significant.

## Results

The patients mean age (± SEM) was 32.8 ± 1.1 years. The mean duration of diabetes (± SEM) was 8.6 ± 0.6 years. The BMI, waist circumference, liver and renal functions tests were within normal limits.

The unemployed patients said they could not find jobs mostly because of the disease. A majority of the patients were working in menial jobs and belonged to the economically weaker sections of society [Table 1].

**Table 1:** Demographic characteristics of the patients*

Characteristic	Number	Percentage
Gender
Male	48	72
Female	19	28
Educational status
Illiterate	14	22
Primary	33	49
Secondary	13	19
Tertiary	7	10
Occupation
Student	-	-
Unemployed	7	11
Housewife	18	26
Employed	42	63
Retired	-	-
Income level
Economically weaker	58	87
Low income	8	12
Middle income	1	1
High income	Nil	Nil

* n = 67

The patients had a mean C-peptide level of 0.19 pmol/ml, indicating that these patients had negligible insulin secretion. The adherence to the insulin regimen as reflected by the plasma insulin level increased from 82 to 86% after the study. Only 52% of the patients had pre-prandial blood glucose levels within normal limits before the intervention. This increased to 60% after the intervention. Similarly, the postprandial blood glucose was within normal limits in 18% of the patients before the intervention, while after the intervention, 31% of the patients were within normal limits. These differences were not statistically significant.

Though overall scores were statistically significant after the intervention [Table 2], four of the items in the knowledge domain did not show any improvement [Table 3], since the scores at the first visit itself were very high.

**Table 2:** Learning outcomes in patients completing a comprehensive monthly educational program on diabetes for a period of six months

	Scores		

Variables (n = 67)	First visit	Last visit	*P* value
Knowledge	10.8 ± 0.3	20.1 ± 0.2	0.0001
Attitude	2.4 ± 0.1	3.3 ± 0.09	0.0001
Practice	17.7 ± 0.3	18.8 ± 0.3	0.0004
Total	30.8 ± 0.5	42.2 ± 0.4	0.0001

Values are mean ± SEM

**Table 3:** Proportion of patients* giving correct responses to the KAP questions before and after the educational intervention

Items under each domain	First visit	Last visit	*P* value
Knowledge			

What is diabetes	99	100	0.3
Two symptoms of diabetes	61	86	0.001
Two complications of diabetes	50	80	0.0004
Dose of Insulin	93	94	0.8
Timing of insulin dose	97	97	1
Consequences of too much insulin	83	97	0.005
Consequences too little insulin	68	93	0.0005
Rotation of injection site	97	99	0.4
Attitude			
Diabetes is a life-long disease	72	92	0.002
Insulin is not a safe drug	58	80	0.005
Maintaining diet in addition to taking insulin	57	82	0.04
Inclination to learn about diabetes from media	49	70	0.01
Practice			
Demonstrated accurately withdrawal of insulin dose	74	80	0.4
Proper storage of insulin vials in home	50	87	<0.0001
Attending diabetic clinic regularly (miss < 3 visits/year)	27	30	0.7
Buy insulin from chemist if insulin is insufficient	9	11	0.7
Alcohol	52	52	1
Cigarette/beedi smoking	50	50	1
Blood sugar testing (3 times/year)	14	19	0.4
Does not exercise but active	76	80	0.6
Avoiding sugar when taking tea/coffee	55	58	0.7
Missing insulin dose (1–2 doses/week)	73	71	0.8

* n = 67

Comparison was made by using test of proportions for dependent groups. The overall mean scores for all domains showed significant improvement, though there was an improvement in only one item of the practice domain after intervention [Table 3]. The reason given by patients for not coming regularly to receive the insulin vials was that they were unable to meet their monthly transportation charges. Ninty-one percent of the patients either reduced their insulin dose or stopped taking insulin if they could not come to receive insulin. There were a small percentage of patients who regularly reduced the insulin dose for the fear of hypoglycemia. However, they never discussed this with the prescribing clinicians.

**Figure 1:**
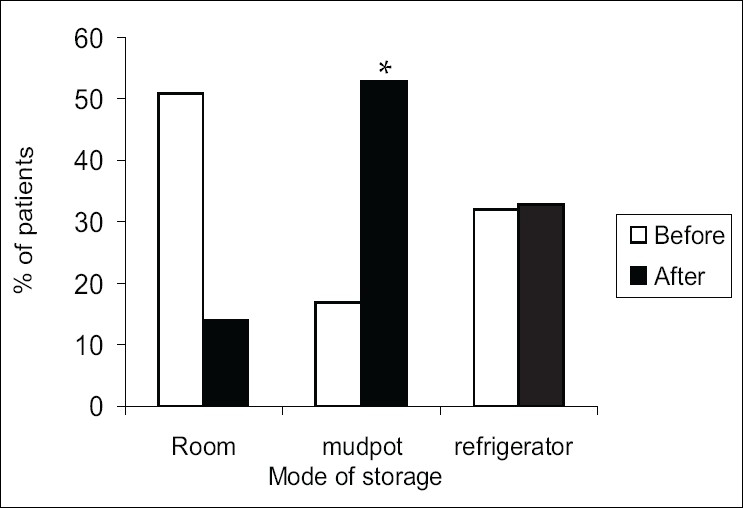
Storage pattern of insulin vials at patients' home before and after intervention, *P < 0.001 using test of proportions when compared to before intervention; (n = 67)

After the intervention, a significant difference was seen in the manner in which patients stored the insulin vials [Figure 1]. Twenty patients stored the vials in a refrigerator at the nearby chemist shop, since they did not have one at home.

## Discussion

The study shows that a planned educational intervention on type-1 diabetics, receiving monthly supplies of insulin free of charge, did not improve the key aspects of the practice component, even though the knowledge and attitude improved. Patients found it difficult to regularly attend the clinic, monitor their blood glucose and take their daily dose of insulin, mainly due to lack of family support and financial constraints. Though blood sugar could be tested free of cost at JIPMER hospital, patients were not in a position to travel in a fasted state from long distances to avail this facility. Since most of these patients were either unemployed or had menial jobs and has to come from far-off places, travelling even once a month strained their meager financial resources. To counter their inability to come regularly, nearly 91% of the patients either reduced their daily insulin dose so that they could adjust with the number of vials dispensed to them for some more days, or did not take insulin at all. The approximate cost of an insulin vial is Rs.160 and only a small percentage of these patients buy their insulin, if they cannot come to get their free monthly supply. Therefore, adherence remained poor despite the improvement in patients' knowledge and attitude. Hence, none of the biochemical markers like blood sugar levels or HbA1c showed any improvement.

Taking into account the fact that most of these patients had only primary school education or were illiterates, their knowledge of the disease was excellent. Their scores were high even before the intervention. This could be due to the long duration of the disease (nearly 8–9 years) in our sample. However, it is the practice component which will make the difference to the management of the disease and this domain did not show a clinically significant improvement, though an overall improvement in scores were seen. Another study conducted in Malaysia with patients from similar background also showed that though knowledge and attitude components scored high, the practice components scored low.[13] In our study, patients readily changed the practice component which can be done without any high financial input, such as storing the vials in a mudpot. It is possible though, that for some of the other items in the practice domain, they were in the contemplation stage of ‘stages of change’ theory where the patient felt that they should improve their self-management skills of diabetes, but there was no committed effort.[6]

The intervention did not improve the adherence to the insulin regimen and even though the post-prandial blood glucose levels decreased significantly from the baseline values, these were far from the normal limits and as such cannot be taken as improvement. However, the decrease in post-prandial blood glucose is a good trend since it is this which contributes significantly to the development of micro and macro vascular diseases.[14]

A vial of human insulin costs between Rs. 130 and 160. The financial burden of issuing free monthly supplies to diabetics is very high. If adherence is improved, the benefit would outweigh the cost involved in providing the drug. However, this study has shown that adherence did not improve and moreover, is unlikely to improve since the reasons are not due to lack of knowledge or attitude. Therefore, issuing the insulin at points nearer to their homes, like at the primary health centers or through the village health workers may have a better impact. Diabetes education and awareness programs and distribution of free supplies of the drug are not enough to improve diabetic control. An awareness of the constraints of the patients to effectively put into practice their knowledge of the disease and addressing those issues is equally important.

This study is limited to patients attending JIPMER hospital and cannot be extrapolated to patients from higher socioeconomic strata and more educated than this sample. However, since large populations in India and in many developing countries belong to this group, health planners should take the lessons learned from this study into account. The study concludes that successful implementation of an educational program coupled with issuing free monthly supplies of insulin may not translate into an improvement in glycemic control or adherence, if the socioeconomic status of the patients is so low that they cannot even find the resources to travel to the hospital on a regular basis.
